# De-pathologizing multiethnic youth: a matched-sample comparison of multiethnic and monoethnic adolescents' psychological health

**DOI:** 10.3389/frhs.2026.1802401

**Published:** 2026-05-04

**Authors:** Hali Kil, Serena Shukla, Amanda Hodges

**Affiliations:** 1 Department of Psychology, Simon Fraser University, Burnaby, BC, Canada; 2 Department of Psychology, BC Children’s Hospital Research Institute, Vancouver, BC, Canada

**Keywords:** mental health, multiethnic, propensity score matching, well-being, youth

## Abstract

**Objective:**

Multiethnic youth are often pathologized in clinical settings, owing to their negative experiences with discrimination, social rejection, and multiethnic identity navigation. While much research suggests that multiethnic youth experience heightened mental health difficulties compared to monoethnic youth, some research suggests that multiethnic youth report positive psychological well-being. The present work aimed to address this inconsistency by comparing mental health and well-being in a sociodemographically matched sample of multiethnic and monoethnic youth.

**Method:**

Participants were 14- to 18-year-old multiethnic (*n* = 309) and monoethnic (*n* = 508) adolescents (M age = 15.6 years; 49.5% girls) of Black, Hispanic, and White backgrounds from 20 US cities. Parent-reported sociodemographic characteristics, collected at the youths' time of birth, and youth self-reported gender and age were used for propensity score matching. MANOVA and *t*-tests were used to compare the matched sample of multiethnic and monoethnic youth on their self-reported mental health (anxiety, depression) and well-being (engagement, perseverance, happiness).

**Results:**

Matched sample comparisons showed that multiethnic youth experienced better mental health on both anxiety and depression symptomology compared to monoethnic youth, although effect sizes were small. No significant differences emerged for well-being. However, trends in comparisons differed by specific ethnic heritage of multiethnic youth and monoethnic youth being compared.

**Conclusions:**

Our findings suggest to caution against pathologizing multiethnic youth as experiencing elevated psychological difficulties compared to monoethnic youth solely based on multiethnic classification. We highlight the need to target sociodemographic factors contributing to psychological health of all youth, regardless of their ethnic identities.

The proportion of youth that belong to multiple ethnic, and corresponding multiple racial and cultural backgrounds—herein referred to as multiethnic youth—has been steadily growing over the past few decades. For example, in the USA, the number of multiethnic youth among all children under 18 years of age rose from 5.6% in 2010 to 15.1% in 2020 (1), showing a three-fold increase over just 10 years. While they have been rising in prominence among the overall youth population, the body of literature on their mental health and well-being is highly contradictory and inconclusive, warranting deeper investigation into the psychological health of this demographic of youth.

Despite being heralded as a symbol of integration of diversity in a society, multiethnic youth are often pathologized in clinical settings and beyond (2–4). These biases may be partially due to the elevated risk for mental health difficulties among multiethnic populations: among 10- to 24-year-old multiethnic youth, estimates of depression are as high as 15.8%, up to three times higher than the global youth estimate of 5.3% to 12.0% of the population (5). Beyond these prevalence statistics, much extant literature points to significantly higher levels of anxiety and depression symptomology and negative mood among multiethnic youth compared to their monoethnic (i.e., single ethnicity) peers (6–9). Even during the recent pandemic, longitudinal changes in anxiety symptoms were much more pronounced among multiethnic 8- to 17-year-olds compared to their monoethnic Asian, Black, and White counterparts (10). Beyond anxiety and depression and turning to general markers of psychological well-being, multiethnic youth have also been found to experience more negative affect and feel less social acceptance and integration compared to monoethnic peers (6, 11).

However, while a wealth of past literature casts a pathologizing perspective on multiethnic youth, there is also some limited evidence that multiethnic youth are faring just as well or sometimes even better than monoethnic youth. For example, among a sample of nationally representative 12- to 18-year-old youth, Milan and Keiley (3) reported that symptoms of depression among multiethnic youth were higher when compared to White monoethnic peers, but lower when compared to minoritized monoethnic peers. Similarly, other studies have found that multiethnic youth report more depression symptoms compared to White monoethnic peers, but fewer compared to Asian, Black, and American indigenous monoethnic peers (6), and similar anxiety symptoms compared to White monoethnic peers, but more compared to Black monoethnic peers (12). With regards to markers of overall psychological well-being, multiethnic youth have reported equivalent levels of global well-being, positive affect, and self-esteem, and higher positive affect and less stress compared to monoethnic peers of diverse backgrounds (11, 13).

The tendency to pathologize multiethnic youth appears to be founded upon assumptions of heightened identity confusion, discrimination from the groups to which they belong, and potential social rejection of their identities (2, 3, 14–17). However, taken together, extant available literature appears to show inconsistencies about the elevated challenges among multiethnic youth when compared to monoethnic youth, challenging this pathologizing perspective. In particular, the specific ethnic backgrounds of both the multiethnic and monoethnic youth under study may be important to examine beyond global differences, lending nuance on which multiethnic youth may be faring better than others in their psychological health. However, with little research attention on the psychological health of multiethnic youth over the years, there is still a need for further examination of psychopathology and well-being in this population.

## The present study

In the present work, we aimed to examine the psychological health of multiethnic youth in relation to their counterparts by addressing two potential confounds in past research that have led to inconsistencies. First, our study compares psychological health between sociodemographically matched samples of multiethnic and monoethnic youth. Based on past literature, multiethnic and monoethnic youth differ in a number of sociodemographic characteristics, including gender and age as well as familial domains such as household income, parental education, parental divorce or separation, and even family employment rates (18–21). Although their consequences may not be entirely direct (and instead, through correlated parenting strategies or altered familial relationships for example) (22, 23), these sociodemographic characteristics are also key contributing factors for risk of psychopathology among youth more generally (24, 25). However, many previous studies comparing mental health outcomes among multiethnic and monoethnic youth have not considered these potential confounds at all or have only considered a limited number of them, leading to potential bias in their findings. We propose that one of the reasons for the inconsistent findings on multiethnic youths' mental health in past literature is due to the omission of potential sociodemographic confounds, and utilize a matching process called propensity score matching (PSM) to address this issue in our study.

Second, our study compares psychological health between multiethnic and monoethnic youth broadly, but takes a nuanced approach in comparing matched samples of specific ethnic backgrounds of youth. For example, rather than examining whether multiethnic youth are generally faring worse or better than monoethnic White and minoritized youth, we examine how psychological health compares across specific backgrounds of multiethnic youth and their paired monoethnic counterparts (e.g., comparing how Black and Hispanic multiethnic youth fare compared to both Black monoethnic peers and Hispanic monoethnic peers). We note that our identification of multiethnic youth is based on parentage, and thus this study focuses on the mental health of multiethnic youth that may not necessarily self-identify as being multiethnic. This is an important point as self-identified multiethnic youth may have different lived experience with developmental processes compared to youth who are multiethnic by parentage only and not by self-identification (26, 27). We propose that such an approach provides nuance on whether certain multiethnic youth may be faring better than those who are minoritized but belong to a single ethnic background, better highlighting the experiences of multiethnic youth that may be different from those of monoethnic minoritized youth.

Thus, overall, the present study sought to compare anxiety and depression symptoms and well-being among multiethnic and monoethnic teenage youth living in the USA, using sociodemographically matched samples and examining nuances in the comparisons by specific ethnic background of multiethnic youth. We focused on the teenage years as this is a critical developmental stage, characterized by a combination of psychological, emotional, and physiological changes (28, 29). Although some youth may flourish during this period, many are susceptible to experiencing well-being and mental health difficulties (30–33). This developmental stage is also a time of elevated identity-related fluctuations, including ethnic, racial, and cultural identities, particularly among youth with multiple ethnic and racial backgrounds (34, 35), who have been shown to experience elevated psychological health risks (3, 17). Understanding this critical period among multiethnic youth provides valuable information on the prevalence of mental health difficulties in and evidence for or against continued psychopathologizing of this population.

## Methods

### Participants & procedures

The present study utilized data from the Future of Families and Child Wellbeing Study (FFCWS) (36). The FFCWS is a longitudinal dataset which includes low-income and socioeconomically marginalized families from 20 large urban cities in the USA with at least one child born between 1998 and 2000. At Wave 1 (baseline or Child Age 0), a total of 4,989 biological parents were interviewed at 75 hospitals. Follow up interviews by phone and in-home assessments were completed when child was 1, 3, 5, 9, and 15 years of age. The current study uses data from Wave 1 (baseline or Child Age 0) and Wave 6 (Child Age 15). Wave 1 data was used to identify multiethnic families and to ensure that our matched sample would reflect similar demographic profiles (see below) and Wave 6 data was used for main analyses as adolescent self-perceived mental health and well-being are particularly important to consider in clinical settings as children reach adolescence (37, 38). All participants gave written and informed consent to participate, and received compensation for participation ($100 for parents, $50 for adolescents).

At Wave 1, biological mothers and fathers reported one ethnic background of four options in the demographic section of the interview: White non-Hispanic, Black non-Hispanic, Hispanic, and other. As all families were biological, children were considered multiethnic when the mother and father each reported a different response to this question. We excluded parents classified by the FFCWS team as “Other” due to the lack of clarity regarding the specific ethnic or ethnic backgrounds included in the category.

Based on this data, a total of *N* = 367 multiethnic children were identified at Wave 1. Among these multiethnic children, only 366 provided self-reported data on mental health and well-being at Wave 6. Of this number, 309 were retained after omitting outliers on our target dependent variables and after participants were automatically removed through listwise deletion on PSM. Adolescents in this final sample were on average 15.6 years of age (SD = 0.8 years), and 49.5% were girls. Most adolescents were Black-Hispanic (41.1%) in background, with fewer numbers of White-Hispanic (36.6%) and Black-White (22.3%) adolescents. At birth of the child, mothers of these adolescents were on average 24.4 years of age (SD = 5.5 years), with the majority married or cohabitating with the child's biological father (55.0%), having completed at least high school education (87.4%), and maintaining an average household income of $60,478.45 per annum (SD = $47,930.79), and employed (77.7%).

#### Propensity score matched monoethnic sample

Given that multiethnic and monoethnic adolescents' mental health and well-being may be explained by a number of demographic characteristics, we matched each multiethnic adolescent to a comparable monoethnic adolescent of each ethnic background using Propensity Score Matching without replacement with the *matching* package (39) in *R* version 4.4.2. Propensity score matching (PSM) is a regression-based matching technique used to compare groups that may differ on several variables, such as demographic variables (40). Matching entails first creating a regression coefficient that represents a propensity score based on the chosen variables. This propensity score is then used to pair a participant from one group with a participant from another group who, based on the chosen variables, has a similar data profile. The results are matched sets of participants that can then be compared. The method is most often used in clinical case-control situations in which a researcher aims more confidently retrospectively analyze the difference between intervention and control group participants when the data were not initially collected with randomized assignment.

We identified a number of demographic characteristics at Waves 1 and 6 that may be associated with adolescent mental health and well-being generally and that have been found to differ across multiethnic and monoethnic samples in past literature. These included mother-reported child gender at Wave 1, and adolescent-reported age as well as mother-reported education, employment status, marital status, and household income at Wave 6 (18, 20, 21, 41). Mothers' education levels were based on four categories: 1 = less than high school, 2 = high school graduate/GED or equivalent, 3 = some college, and 4 = college graduate or graduate school and was measured using these four categories. Mothers' employment status and marital status (with either biological parent or new partner) were both measured dichotomously (0 = no; 1 = yes). Finally, mother-reported household income was identified in the following categories: 1 = <$5,000, 2 = $5,001–$10K, 3 = $10,001–$15K, 4 = $15,001–$20K, 5 = $20,001–$25, 6 = $25,001–30K, 7 = $30,001–$40K, 8 = $40,001–$60K, 9 = $>60K. Father-reported demographics were initially included in PSM, but were later eliminated (except for fathers' ethnicity) due to high rates of missing data that resulted in a substantially reduced sample size due to automatic listwise deletion in PSM protocol.

As each multiethnic adolescent represented two different ethnic backgrounds that could be matched to a monoethnic adolescent (e.g., a Black-Hispanic adolescent could be matched to both a monoethnic Black or monoethnic Hispanic adolescent), PSM was conducted a total of six times for approximately double the matched sample size of Black, Hispanic, and White monoethnic adolescents to multiethnic adolescents. Total sample sizes and descriptive statistics on the selected demographic variables for multiethnic and monoethnic adolescents before and after PSM are depicted in Table 1. After PSM, a total of 508 matched monoethnic youth were retained for further analyses.

**Table 1:** Participant demographics by ethnoracial background, Pre and post propensity score matching.

Variable	Multiracial Adolescents	Monoracial Adolescents					
		Black		Hispanic		White	
		Pre-PSM	Post-PSM	Pre-PSM	Post-PSM	Pre-PSM	Post-PSM
*N*	309	1,640	194	694	206	578	165
Adolescent age [M (SD)]	15.62 (.76)	15.62 (.73)	15.65 (.73)	15.66 (.86)	15.75 (.88)	15.43 (.70)	15.46 (.71)
Proportion of boys (%)	50.49	51.60	51.03	51.70	49.51	52.80	55.20
Household income in USD [M (SD)]	60,478.45 (47,930.79)	44,979.89 (38,847.15)	49,083.78 (38,384.82)	49,668.90 (46,045.22)	58,046.14 (42,902.33)	1,09,623.11 (93,244.97)	69,722.56 (48,310.16)
Cohabitating or married (%)	55.00	43.65	47.94	66.82	55.83	75.41	65.45
High school educated or higher (%)	87.34	85.44	85.57	62.20	84.47	93.02	90.91
Employed (%)	77.70	69.43	73.71	68.75	78.64	73.52	76.97

PSM, Propensity score matching.

### Measures

#### Depression

Adolescents reported on their symptoms of depression using five items adapted from the original 20-item version of the Center for Epidemiologic Studies Depression Scale (CES-D) (42),: “I feel I cannot shake off the blues, even with help from my family and my friends”, “I feel sad”, “I feel happy”, “I feel life is not worth living”, and “I feel depressed”. Adolescents responded to each statement as they experienced it over the past four weeks using a four-point scale from *1* = strongly agree to *4* = strongly disagree. Items (except “I feel happy”) were reverse coded and averaged so that larger values indicated higher depression. Scores were only considered valid if adolescents completed at least two-thirds of items. The original CES-D displayed high internal consistency in a sample of ethnically diverse adolescents in grades 7 through 12 (i.e., aged 13 to 18 years) living in the USA (43). In the present sample, interitem consistency as indicated by Cronbach's alpha (*α*) was *α* = 0.69 for multiethnic adolescents and *α* = 0.76 for monoethnic adolescents.

#### Anxiety

Adolescent reported on their anxiety levels using six items drawn from the Brief Symptom Inventory (BSI-18) anxiety subscale (44): “I have spells of terror or panic”, “I feel tense or keyed up”, “I get suddenly scared for no reason”, “I feel nervous or shaky inside”, “I feel fearful”, and “I feel restless, I can't sit still”. Adolescents responded to each statement as they experienced it over the past four weeks using a four-point scale from *1* = strongly agree to *4* = strongly disagree. Items were reverse coded and averaged so that larger values indicated higher anxiety. Scores were only considered valid if adolescents completed at least two-thirds of items. The original BSI-18 displayed good internal consistency in a past study of low-income African American adolescents between 13 and 22 years old living in the USA (45). In the present sample, interitem consistency was *α* = 0.69 for multiethnic adolescents and *α* = 0.74 for monoethnic adolescents.

#### Well-Being

Adolescents reported on their well-being using the EPOCH (Engagement, Persistence, Optimism, Connectedness, Happiness) Measure of Adolescent Well-being (46). This measure includes 20 items that divide into the following five subscales that model the PERMA (Positive Emotions, Engagement, Relationships, Meaning, and Accomplishment), model of well-being: engagement (e.g., “I get so involved in activities that I forget about everything else”), perseverance (e.g., “Once I make a plan to get something done, I stick to it”), optimism (e.g., “I believe that things will work out, no matter how difficult they seem”), connectedness (e.g., “I have friends that I really care about”), and happiness (e.g., “I am a cheerful person”). Adolescents responded to each statement as they experienced it over the past four weeks using a four-point scale from *1* = strongly agree to *4* = strongly disagree. Items were reverse coded and averaged for each subscale so that larger values indicated higher well-being. Scores for each subscale were considered valid only if adolescents completed at least two-thirds of the corresponding items. The EPOCH displayed good internal consistency in a sample of 10 to 18 year old adolescents in the USA and Australia (46). In the present sample, interitem consistency of the full EPOCH was *α* = 0.70 for multiethnic adolescents and *α* = 0.78 for monoethnic adolescents. Interitem consistency for each subscale of the EPOCH was as follows: engagement (*α* = 0.58 for multiethnic adolescents and *α* = 0.55 for monoethnic adolescents), perseverance (*α* = 0.64 for multiethnic adolescents and *α* = 0.70 for monoethnic adolescents), optimism (*α* = 0.36 for multiethnic adolescents and *α* = 0.57 for monoethnic adolescents), connectedness (multiethnic adolescents: *α* = 0.41, monoethnic adolescents *α* = 0.55), and happiness (*α* = 0.63 for multiethnic adolescents and *α* = 0.75 for monoethnic adolescents). As the interitem reliability of the optimism and connectedness subscales fell below .50—i.e., the minimal threshold for scales with fewer than 10 items (47, 48)—we dropped these scores from all further analyses.

### Analysis

We conducted a single MANOVA to examine whether the overall samples of multiethnic and monoethnic adolescents differed in their self-reported depression, anxiety, and well-being. In addition, we conducted a series of paired *t*-tests to examine whether these comparisons were consistent among different ethnic backgrounds of matched multiethnic and monoethnic adolescents. As each multiethnic adolescent subsample was exactly matched to an equally sized monoethnic adolescent subsample, a total of six paired *t*-tests were conducted for each outcome variable of interest, with the following pairings: Black-White multiethnic and Black monoethnic adolescents, Black-White multiethnic and White monoethnic adolescents, Black-Hispanic multiethnic and Black monoethnic adolescents, Black-Hispanic multiethnic and Hispanic monoethnic adolescents, Hispanic-White multiethnic and Hispanic monoethnic adolescents, and Hispanic-White multiethnic and White monoethnic adolescents. Given multiple tests on the same outcome variables, we manually corrected our *p*-value cutoff to *p* *=* .008 (i.e., .05 traditional cutoff divided by 6 tests). No covariates were included in our comparisons, as the prior step of PSM eliminated any lingering differences in sociodemographic characteristics across groups being compared.

## Results

Means, standard deviations, and correlations for all retained dependent variables are depicted in Table 2. All variables were correlated in the expected directions—that is, positive correlations for most constructs except for depression and anxiety—confirming the use of a MANOVA rather than multiple ANOVAs for testing overall group differences between multiethnic and monoethnic adolescents.

**Table 2:** Descriptive statistics and correlations for variables of interest.

Variable	*M*	*SD*	1	2	3	4	5
1. Depression	1.56	.56					
2. Anxiety	1.79	.61	.62				
3. Total Well-being	3.44	.29	−.47	−.18			
4. Engagement	2.99	.59	.10	.26	.51		
5. Perseverance	3.41	.47	−.30	−.23	.65	.06	
6. Happiness	3.59	.46	−.64	−.32	.73	.13	.37

All correlations significant at *p* < .01.

**Figure 1:**
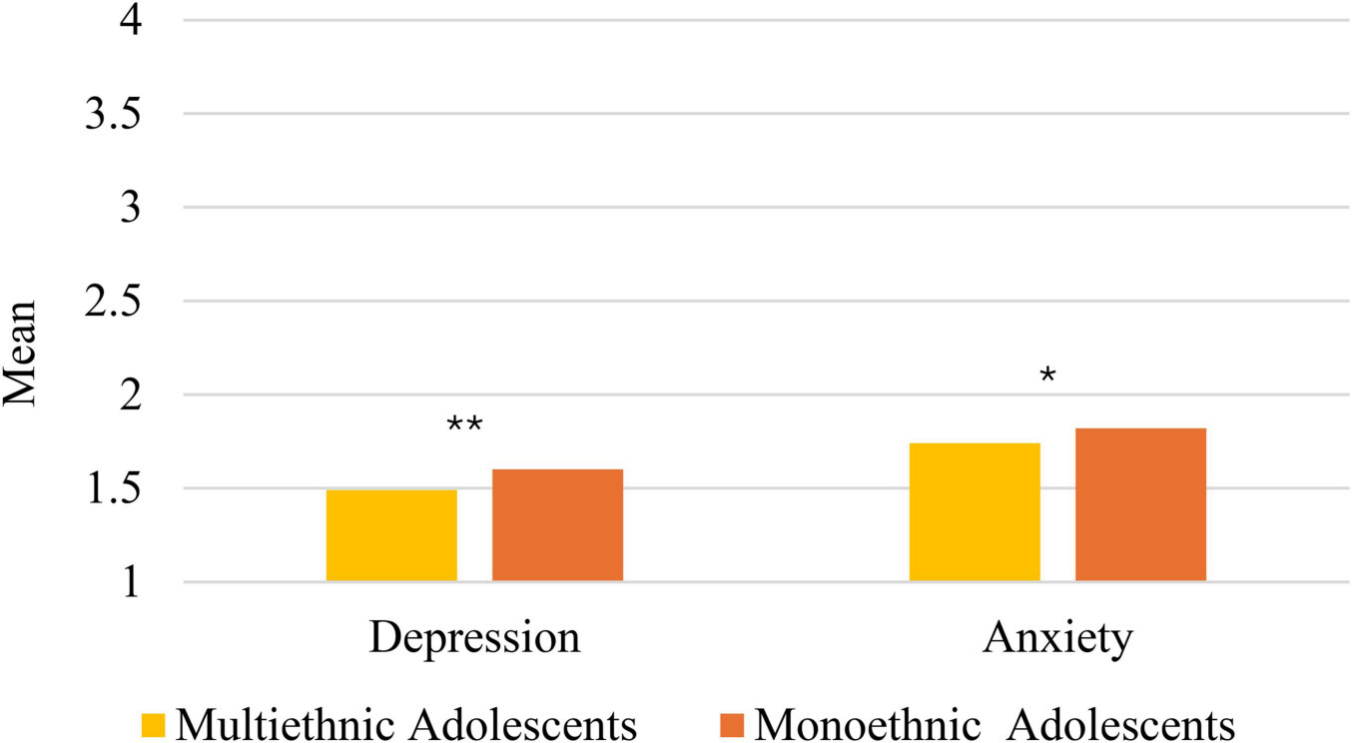
Depression and anxiety in multiracial vs. monoracial adolescents. Higher values indicate higher levels of anxiety and depression. * *p* < 0.05 ** *p* < 0.01.

In our main analyses, significant group differences were found for depression, *F*(1,871) = 6.90*, p* = .009, partial *η*^2^ = .008, and anxiety, *F*(1,871) = 3.91, *p* = .048, partial *η*^2^ = .004, with effect sizes indicating a small effect (58). As depicted in Figure 1, results indicated that depression and anxiety levels were significantly lower among multiethnic (*M* = 1.49; SD = .49 for depression, *M* = 1.74; SD = .56 for anxiety) than among monoethnic adolescents (*M* = 1.60; SD = .60 for depression, *M* = 1.82; SD = .64 for anxiety). Total well-being, and subscale well-being scores of engagement, perseverance, and happiness scores did not significantly differ across multiethnic and monoethnic youth, *F*s < 1.61, *p*s > 0.21.

### Subgroup analyses

**Figure 2:**
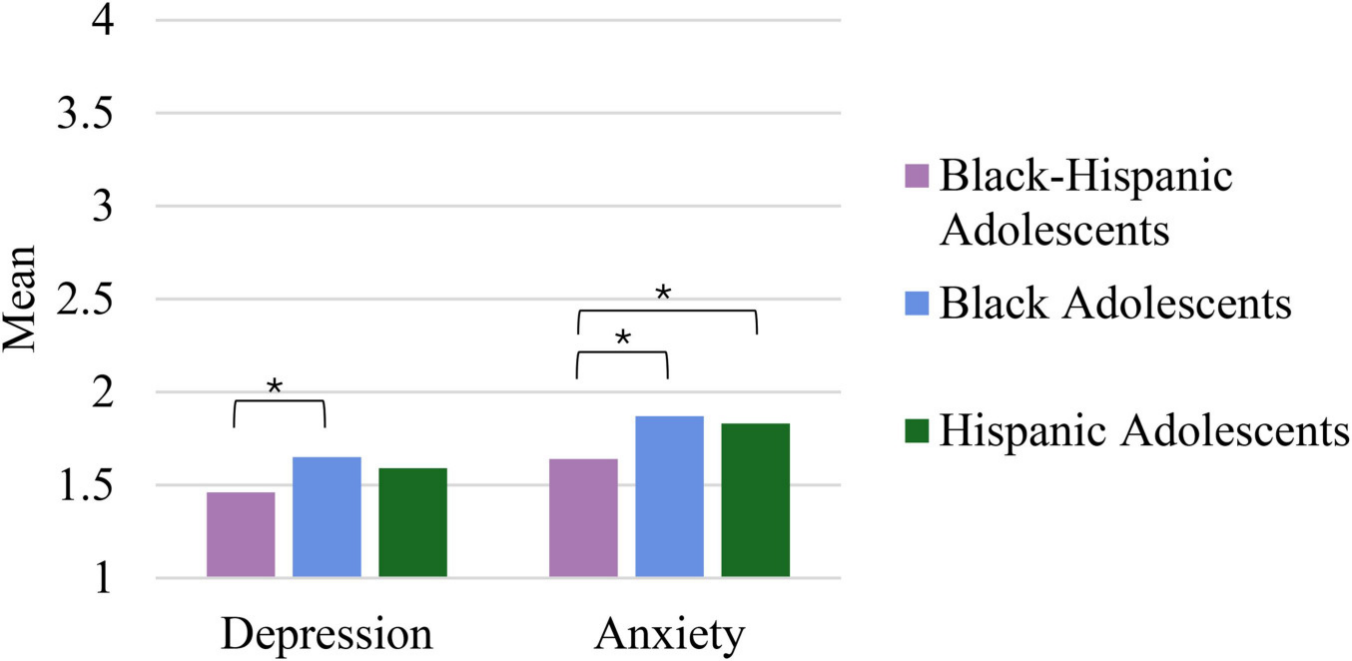
Depression and anxiety across subgroups. Higher values indicate higher levels of anxiety and depression. * *p* < 0.008.

For depression symptoms, paired *t*-tests revealed significant differences only between Black-Hispanic (*M* = 1.46; SD = .46) and Black adolescents (*M* = 1.65; SD = .65), *t*(126) = −2.81, *p* = .003, *d* = −.249. That is, as depicted in Figure 2, Black-Hispanic multiethnic adolescents reported significantly less depression symptoms compared to Black monoethnic adolescents. No other paired comparisons were significant, *t*s < −2.14 *p*s > .018.

For anxiety symptoms, paired *t*-tests revealed significant differences between Black-Hispanic (*M* = 1.64; SD = .49) and Black (*M* = 1.87; SD = .70) adolescents, *t*(126) = −3.277, *p* < .001, *d* = −.291, and between Black-Hispanic and Hispanic (*M* = 1.83; SD = .61) adolescents, *t*(126) = −2.729, *p* = .004, *d* = −.242. That is, as depicted in Figure 2a, Black-Hispanic multiethnic adolescents reported significantly less anxiety symptoms compared to Black or Hispanic monoethnic adolescents. No other paired comparisons were significant, *t*s < 1.18, *p*s > .068.

For well-being, no paired comparisons were significant for overall well-being, *t*s < 2.22, *p*s > .015, for perseverance, *t*s < 2.31, *p*s > .011, or for happiness, *t*s < 1.61, *p*s > .056. For engagement, paired *t*-tests revealed significant differences only between Black-Hispanic (*M* = 2.88; SD = .65) and Black adolescents (*M* = 3.12; SD = .60), *t*(126) = −3.09, *p* = .001, *d* = −.274. That is, Black-Hispanic multiethnic adolescents reported significantly less engagement compared to Black monoethnic adolescents. No other paired comparisons were significant for engagement, *t*s < 2.34, *p*s > .010.

## Discussion

The present study sought to assess anxiety and depression symptoms and psychological well-being among multiethnic youth in comparison to a sociodemographically matched sample of monoethnic youth using a large, nationally representative dataset from the USA. Contrary to much of the existing literature e.g. (2, 6), we found that multiethnic youth experienced lower levels of anxiety and depression compared to monoethnic youth, while well-being levels did not significantly differ across groups. Our findings echo some past findings that have similarly found better or similar psychological health among multiethnic youth relative to monoethnic youth e.g. (12, 49). It has been suggested that sociodemographic characteristics, such as family income and parent marital status, play pivotal roles for youth mental health due to the sequalae of potentially dysregulated family dynamics and lack of parent involvement that may result in at-risk families (50). In line with these perspectives, our findings implicate the importance of considering sociodemographic profiles of multiethnic youth presenting with mental health difficulties. Specifically, rather than generally pathologizing multiethnic youth due to their ethnic background and identity, consideration of familial and household demographic factors may be helpful to more accurately understand the psychological health of multiethnic youth in context and in relation to their monoethnic counterparts, at least based on ethnic classification.

Our findings also emphasize that certain ethnic classifications of multiethnic youth may be particularly protected from experiencing poor psychological health, compared to matched monoethnic counterparts. In particular, Black-Hispanic multiethnic youth in our study reported fewer anxiety and depression symptoms compared to Black and Hispanic monoethnic youth, while no other subgroup analyses revealed similar findings. Dual minoritized youth such as Black-Hispanic youth have been thought to experience poorer mental health outcomes compared to monoethnic youth due to potential dual rejection, discrimination, and social exclusion from both of their racialized background communities (16, 17, 51). Contradicting these perspectives, our findings point to a potentially positive effect of dual minoritized multiethnic status on psychological health, echoing a handful of evidence suggesting that dual minoritized youth have an advantage in being able to find social connection and community in highly diverse community settings due to their capacity for cultural sensitivity and understanding (52–54). It is also possible that these contradicting findings are due to the ethnic classification approach (rather than self-identification) used in this study to identify multiethnic youth; however, further research is needed to ascertain these distinctions and implications for psychological health.

Although the findings of this study contribute to existing literature by casting a positive, de-pathologizing light on multiethnic youths' psychological health, it also has several limitations. First, while novel to this topic, our selection of sociodemographic variables for propensity score matching was limited to familial and parental variables that have been previously found to differ across multiethnic and monoethnic families (59). Other notable sociodemographic characteristics that may influence youth development, such as neighbourhood quality, food insecurity, and multigenerational household status (i.e., grandparent involvement), were not included in our study due to lack of such data and thus not accounted for in our matched samples. In particular, assessing broader contextual factors such as multiethnic representation in the youths' neighbourhoods and school climate regarding race and racial issues may shed further light on the nuanced experiences of multiethnic youth. Our study also examined only youth-reported mental health and well-being outcomes, which may be considered a limitation due to single-responder bias. While we acknowledge the importance of multi-informant methods to assess mental health outcomes, we also note that youths' self-reports of mental health are often more informative in gauging their self-perceived longer-term outcomes compared to other sources during adolescence (55).

Our study is also based on an existing large-scale dataset that oversampled Black and Hispanic families and thus does not necessarily reflect the level of ethnoracial diversity among multiethnic families today. For example, recent statistics suggest that Asian-multiethnic individuals may be most likely to self-identify as multiethnic, and that the majority (up to 50%) of USA's multiethnic population consists of White-American and Indigenous multiethnic individuals, including Alaskan Native and American Indigenous multiethnic youth (56). As this existing dataset did not include youth of diverse Asian backgrounds, further research sampling such groups is needed to extrapolate the meaningfulness of our matched comparisons for diverse multiethnic youth populations, such Pacific Islander and Native Hawaiian multiethnic youth. However, with a further diversified sample, concerns surrounding heterogeneous experiences arise—that is, well-being or mental health may be interpreted differently by youth of different multiethnic backgrounds. This may partly explain the lower reliability of the well-being measures among multiethnic relative to monoethnic youth, suggesting between-item heterogeneity in the interpretation of the well-being questions. In general, our well-being subscales had lower than typical interitem reliability, and future work may explore the nuances of well-being among multiethnic youth to determine if some descriptors may more optimally describe their experiences.

An additional consideration is the use of classification of multiethnic background of youth based on parentage, which may obfuscate the lived experiences of self-identifying vs. non-self-identifying multiethnic youth. Future research examining mental health and well-being in the context of multiethnic identity centrality, integration, and conflict (57) would provide more nuance in understanding their developmental outcomes. As ethnicity and culture are highly related constructs, assessing the role of bicultural or multicultural identity and engagement on psychological health may also be a promising avenue for future work.

Lastly, our findings highlight a cross-section of multiethnic youth outcomes at adolescence, rather than casting a longitudinal trajectory lens. Once again, this was a dataset limitation, as there was not data collected on youth self-reported mental health prior to adolescence. Future longitudinal research mapping multiethnic youths' mental health across development would provide critical information on potential long-term change and stability in mental health among multiethnic youth. Incorporating other variables in such longitudinal studies may also provide explanations of mechanisms underlying the relatively more positive mental health outcomes that multiethnic youth may experience compared to monoethnic youth.

## Conclusion

Despite these limitations, the present findings support a positive lens on multiethnic youths' mental health, starkly contrasting the large body of research that suggests multiethnic youth face higher risk for and experience with psychological difficulties. While these findings are based on classification of multiethnic background based on parent ethnicity and race, they still encourage caution when serving multiethnic youth, turning clinical attention towards potential sociodemographic characteristics that may drive historically assumed mental health discrepancies between multiethnic and monoethnic youth. With a growing number of multiethnic individuals across many globalized societies including the USA, a more nuanced and less stigmatizing understanding of multiethnic youths' experiences is needed to best care for their mental health in clinical settings. The present work may be a first step in this direction, encouraging clinical consideration of demographic and contextual factors that may contribute to multiethnic youths' mental health outcomes.

## Data Availability

The datasets presented in this study can be found in online repositories. The names of the repository/repositories and accession number(s) can be found below: https://pop.princeton.edu/.
